# Comparative Analyses of Plastid Sequences between Native and Introduced Populations of Aquatic Weeds *Elodea canadensis* and *E. nuttallii*

**DOI:** 10.1371/journal.pone.0058073

**Published:** 2013-04-19

**Authors:** Tea Huotari, Helena Korpelainen

**Affiliations:** Department of Agricultural Sciences, University of Helsinki, Helsinki, Finland; University of Gottingen, Germany

## Abstract

Non-indigenous species (NIS) are species living outside their historic or native range. Invasive NIS often cause severe environmental impacts, and may have large economical and social consequences. *Elodea* (Hydrocharitaceae) is a New World genus with at least five submerged aquatic angiosperm species living in fresh water environments. Our aim was to survey the geographical distribution of cpDNA haplotypes within the native and introduced ranges of invasive aquatic weeds *Elodea canadensis* and *E. nuttallii* and to reconstruct the spreading histories of these invasive species. In order to reveal informative chloroplast (cp) genome regions for phylogeographic analyses, we compared the plastid sequences of native and introduced individuals of *E. canadensis.* In total, we found 235 variable sites (186 SNPs, 47 indels and two inversions) between the two plastid sequences consisting of 112,193 bp and developed primers flanking the most variable genomic areas. These 29 primer pairs were used to compare the level and pattern of intraspecific variation within *E. canadensis* to interspecific variation between *E. canadensis* and *E. nuttallii*. Nine potentially informative primer pairs were used to analyze the phylogeographic structure of both *Elodea* species, based on 70 *E. canadensis* and 25 *E. nuttallii* individuals covering native and introduced distributions. On the whole, the level of variation between the two *Elodea* species was 53% higher than that within *E. canadensis*. In our phylogeographic analysis, only a single haplotype was found in the introduced range in both species. These haplotypes H1 (*E. canadensis*) and A (*E. nuttallii*) were also widespread in the native range, covering the majority of native populations analyzed. Therefore, we were not able to identify either the geographic origin of the introduced populations or test the hypothesis of single versus multiple introductions. The divergence between *E. canadensis* haplotypes was surprisingly high, and future research may clarify mechanisms that structure native *E. canadensis* populations.

## Introduction

Non-indigenous species (NIS) are species living outside their historic or native range [1]. Invasive NIS often cause severe environmental impacts [2]–[4], and may have large economical and social consequences [2], [3], [5], [6]. Human activities, such as agriculture, aquaculture, recreation and transportation promote both intentional and accidental spread of NIS [7] and the rate of invasions by NIS is accelerating [8]–[10]. Freshwater systems are considered particularly sensitive to invasions for several reasons [11]. Aquatic environments are homogeneous on a large spatial scale and, therefore, aquatic plants can survive and establish more easily outside their native geographic range. Furthermore, aquatic environments are more difficult to monitor, and early detection of submerged species is seldom possible. In addition, water is an effective vector of propagules allowing dispersal over long distances [12], [13].

In order to control the dispersal and effects of invasive species it is important to know the origin of invasive taxa, the size of the introduction, the level of genetic variation compared to the native range, and whether multiple introductions have occurred. Molecular genetic methods have provided new tools to answer these questions [14]–[16]. Molecular markers may also help in early detection of invaders and, therefore, in preventing new invasions [17]. The loss of genetic variation due to the founder effect is a frequent feature in introduced populations compared to the diversity found across the native range [5], [18]–[20]. This combined with multiple introductions and adaptation to a new environment may result in rapid genetic changes in introduced populations [20], [21], enhancing genetic differentiation between native and introduced populations [22] and increasing genetic diversity in individual populations in the invaded region [23], [24].

The gene order and content of cp genomes are generally highly conserved [25] and the substitution rate in cpDNA is much lower than that in the nuclear DNA in plants [26], [27]. Maternally inherited cpDNA is transmitted only through seeds and, therefore, it has less potential for gene flow than nuclear genes, which are spread also by pollen dispersal. Consequently, genetic variation in the cp genome is often more geographically structured than that in the nuclear genome, making chloroplasts valuable sources of genetic markers for intraspecific phylogeographic analyses [28]–[31]. Despite limited variation, cpDNA data have been successfully analyzed and used to trace the origin and biogeographical history of many invasive plants [15], [17], [32]–[38]. Several loci useful for phylogeographic studies have been discovered and exploited since the pioneering work in 1991 [39]; however, many polymorphic loci still remain undetected. The non-coding cp genome regions widely used in phylogenetic and phylogeographic studies might actually be among the least variable ones, whereas the most variable regions are rarely exploited [40], [41]. The number of completely sequenced cp genomes is growing rapidly [28], and lower sequencing costs enable genome-wide intraspecific comparative studies to help detect the most variable genomic regions.

*Elodea* (Hydrocharitaceae) is a New World genus with at least five submerged aquatic angiosperm species living in fresh water environments. The native range of *E. canadensis*, *E. nuttallii* and *E. bifoliata* is temperate North America, while *E. potamogeton* and *E. callitrichoides* are native to South America [32]. In the present paper, we analyzed the phylogeographical cpDNA variation of two invasive species *E. canadensis* and *E. nuttallii*, which have been introduced to other continents [42]. *Elodea canadensis* was brought to Europe in 1836, first to the UK [43], and at present it is widespread in the whole Europe. It was introduced to New Zealand in 1868 [44], to Australia in 1931 [45], and it has been considered a noxious weed also in many regions of Asia and Africa [46]. *Elodea nuttallii* was reported in Europe in 1939, first in Belgium [47]. It has not yet been found in the northernmost Europe, but it is likely to spread to new areas with a high risk of being invasive [47], [48]. The species was introduced to Japan in 1961 [49] and to China in 1980 [50], while it has not yet been found in Australia or New Zealand. Both *Elodea* species were reputedly brought to Europe as aquarium plants or with timber [42]. Once in Europe, water currents and birds have spread these plants locally, while botanists and botanic gardens were responsible for their long distance dispersal [42], [51]. Vegetative reproduction by fragmentation or specialized buds dominates in both native and introduced populations, and in introduced populations sexual reproduction is extremely rare or totally absent [52], [53]. Both species grow aggressively and form dense stands making the recreational use of lakes difficult. These mass occurrences may change the balance of lake and river ecosystems in many ways, *e.g.* by outcompeting native species, changing the pH and nutrient levels and reducing the oxygen concentrations of water column [46], [47], [54].

Both *E. canadensis and E. nuttallii* show a wide range of morphological variation and they are difficult to identify [33], [55]. These species have been successfully discriminated by sequencing the nuclear ITS region [56]. However, ITS has proved to be problematic for several reasons, such as the existence of extensive sequence variation in several plant genomes [57]. Therefore, additional molecular identification tools would be valuable in monitoring risky species and in early detection of new invasions. In this study we compared the plastid sequences of native and introduced individuals of *E. canadensis*. We determined the distribution and location of the most variable regions between the two plastid sequences, and developed PCR-based molecular markers covering these regions. We used these markers to reveal the cpDNA variation in *E. canadensis* and *E. nuttallii* within native and introduced ranges, and tested the hypothesis that the genetic patterns of *E. canadensis* and *E. nuttallii* are more uniform in the introduced range due to a single introduction event. The aims of this study were to 1) survey unexplored areas of the cp genome suitable for phylogeographic analyses and for species discrimination between *E. canadensis and E. nuttallii*, 2) provide detailed genetic data of cpDNA variation within the native and introduced ranges of *E. canadensis* and *E. nuttallii* to test the hypothesis that the genetic patterns are more uniform in the introduced range due to a single introduction event, and 3) survey the geographical distribution of the cpDNA haplotypes across the invasions in order to reconstruct the spreading histories of *E. canadensis* and *E. nuttallii*.

## Materials and Methods

### Plant materials

**Figure 1:**
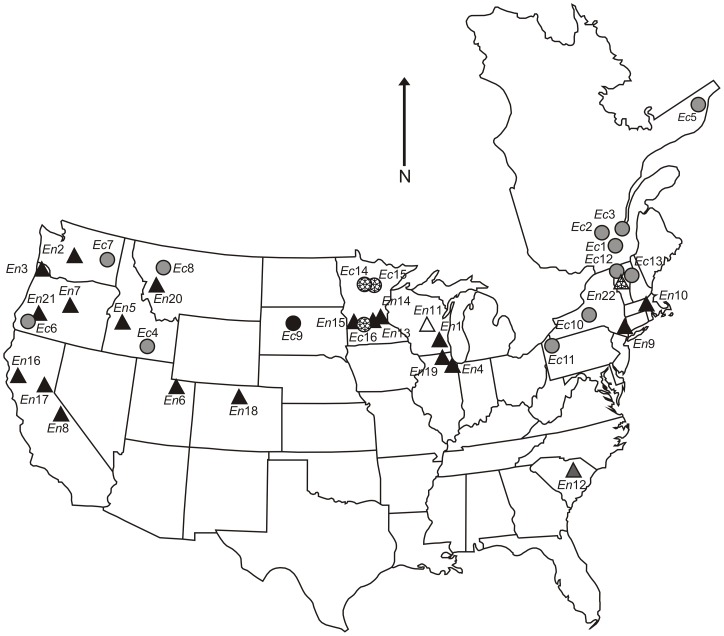
Map of populations sampled in the native range. Sampled populations and distribution of chloroplast DNA haplotypes of *Elodea canadensis* (circles) and *E. nuttallii* (triangles) in the United States of America and Canada. Site numbers correspond to those in Table 1, and shapes, shades and patterns correspond to the haplotypes in Figure 3.

**Figure 2:**
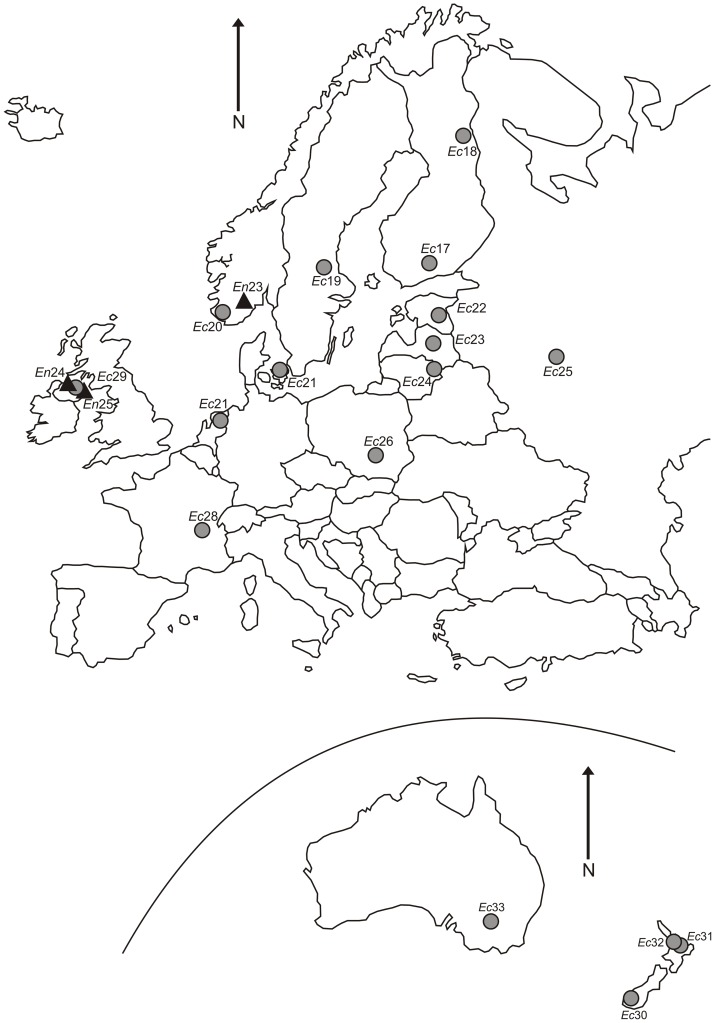
Map of populations sampled in the introduced range. Sampled populations and distribution of chloroplast DNA haplotypes of *Elodea canadensis* (circles) and *E. nuttallii* (triangles) in Europe, New Zealand and Australia. Site numbers correspond to those in Table 1, and shapes and shades correspond to the haplotypes in Figure 3.

The plant material for *E. canadensis* cpDNA extraction and cp genome sequencing was obtained from a native population in Minnesota, the United States of America (N47°14.978′, W95°14.750′) (Site number *Ec*14 in Table 1 and Figure 1). For sending the fresh plant material from the United States to Finland, a phytosanitary certificate was obtained from the United States Department of Agriculture. Leaf material for phylogeographic analyses was collected from 24 *E. canadensis* populations: seven from North America, 13 from Europe, three from New Zealand and one from Australia (Table 1, Figures 1 and 2). Leaf material was also collected from 13 *E. nuttallii* populations: ten from North America and three from Europe (Table 1, Figures 1 and 2). One to three samples were collected from each population. No specific permits were required for collecting the plant material and the field studies did not involve endangered or protected species. Moreover, herbarium specimens collected from the native range and stored at three herbaria (see Table 1) were included in the analyses. Nine herbarium specimens represented *E. canadensis* (five from the United States of America and four from Canada), while 12 represented *E. nuttallii* (all from the United States of America). Species identification was verified by sequencing the nuclear ITS region of each specimen.

**Table 1:** List of *Elodea canadensis* and *E. nuttallii* specimens used in this study and associated haplotypes based on the observed sequence differences within nine combined chloroplast genome regions (total 4072 bp).

Species	Site name	Site code	Collectors and voucher number of herbarium specimens	N	Haplotype
*Elodea canadensis*					
Herbarium specimens from the native range*					
	River Nicolet Sud-Ouest, QC, Canada	*Ec*1	S. Brisson (H1250456)^1^	1	H1
	Saint-Maurice, QC, Canada	*Ec*2	M. Blondeau (H1673242)^1^	1	H1
	Saint Augustin de Desmaures, QC, Canada	*Ec*3	M. Blondeau (H1580693)^1^	1	H1
	Snake River, ID	*Ec*4	R.R. Halse (OSC174966)^2^	1	H1
	St. Augustin county, QC, Canada	*Ec*5	Gravel and J.C. Tessier (OSC136046)^2^	1	H1
	Rogue River, OR	*Ec*6	R.R. Halse (OSC159978)^2^	1	H1
	Windmill Pond, WA	*Ec*7	R. Bursik (NY)^3^	1	H1
	Swan River, MT	*Ec*8	M. Mooar (NY)^3^	1	H1
	Unnamed Lake, SD	*Ec*9	K. Sletten (OSC175763)^2^	1	H2
Fresh specimens from the native range*					
	Chenango Lake, NY	*Ec*10		3	H1
	Lake Pleasant, PA	*Ec*11		1	H1
	Mud Creek, VT	*Ec*12		3	H1
	Halls Lake, VT	*Ec*13		1	H1
	Sucker creek, MN	*Ec*14		3	H3
	Mississippi River, MN	*Ec*15		3	H3
	Otter Lake, MN	*Ec*16		1	H3
Fresh specimens from the introduced range					
	Ditch Nurmijärvenoja, Finland	*Ec*17		1	H1
	Lake Talvijärvi, Finland	*Ec*18		3	H1
	River Österdalälven, Sweden	*Ec*19		3	H1
	Skas-Heigre canal, Norway	*Ec*20		3	H1
	Pølå, near Hillerød, Denmark	*Ec*21		3	H1
	Saadjärv, Estonia	*Ec*22		3	H1
	Lake Juveris, Latvia	*Ec*23		3	H1
	Lake Drūkšiai, Lithuania	*Ec*24		3	H1
	Moscow district, Russia	*Ec*25		1	H1
	River Nida, Poland	*Ec*26		3	H1
	Peize, The Netherlands	*Ec*27		3	H1
	Rhone River, France	*Ec*28		3	H1
	Lake near Helen's Bay, Northern Ireland	*Ec*29		2	H1
	Lake Monowai, New Zealand, South Island	*Ec*30		3	H1
	Lake Tarawera, New Zealand, North Island	*Ec*31		3	H1
	Lake Rotorua, New Zealand, North Island	*Ec*32		3	H1
	Northern Victoria, Australia	*Ec*33		3	H1
*Elodea nuttallii*					
Herbarium specimens from the native range*					
	Lake Beulah, WI	*En*1	J. Meriläinen (H1043562)^1^	1	A
	Klickitat River, WA	*En*2	R.R. Halse (OSC175193)^2^	1	A
	Lewis & Clark National Historic Park, OR	*En*3	Reich and Schull (OSC223622)^2^	1	A
	Hidden Lake Forest Preserve, IL	*En*4	W. Hess, K. Weis and N. Stoynoff (NY)^3^	1	A
	Boise River, ID	*En*5	B. Ertter, S. Richards Harris and J.W. Grimes (NY)^3^	1	A
	Sheep Creek Bay, UT	*En*6	S. Goodrich (NY)^3^	1	A
	Deschutes River, OR	*En*7	J. Mastroguiseppe, G. Milano and K. Cook (NY)^3^	1	A
	Owens River, CA	*En*8	M. Honer (NY)^3^	1	A
	Bronx River, NY	*En*9	D. Atha (NY)^3^	1	A
	Connecticut River, MA	*En*10	H.E. Ahles and R. Paul (NY)^3^	1	A
	Tarrant Lake, WI	*En*11	J. Meriläinen (H1043561)^1^	1	B
	Columbia, SC	*En*12	J.B. Nelson (H1741249)^1^	1	C
Fresh specimens from the native range*					
	Square Lake, MN	*En*13		1	A
	Lake Minnetonka, MN	*En*14		1	A
	Wood Lake, MN	*En*15		1	A
	Lake Hydrilla, CA	*En*16		1	A
	Nevada county, CA	*En*17		1	A
	Grand Lake, CO	*En*18		1	A
	Hastings Creek, IL	*En*19		1	A
	Missoula, Buckhouse Bridge, MT	*En*20		1	A
	Diamond Lake, OR	*En*21		1	A
	Lake Champlain, VT	*En*22		1	D
Fresh specimens from the introduced range					
	Lake Bjaarvatn, Norway	*En*23		1	A
	River Bann, Northern Ireland	*En*24		1	A
	Lady Dixon Park, Northern Ireland	*En*25		1	A

**Figure 3:**
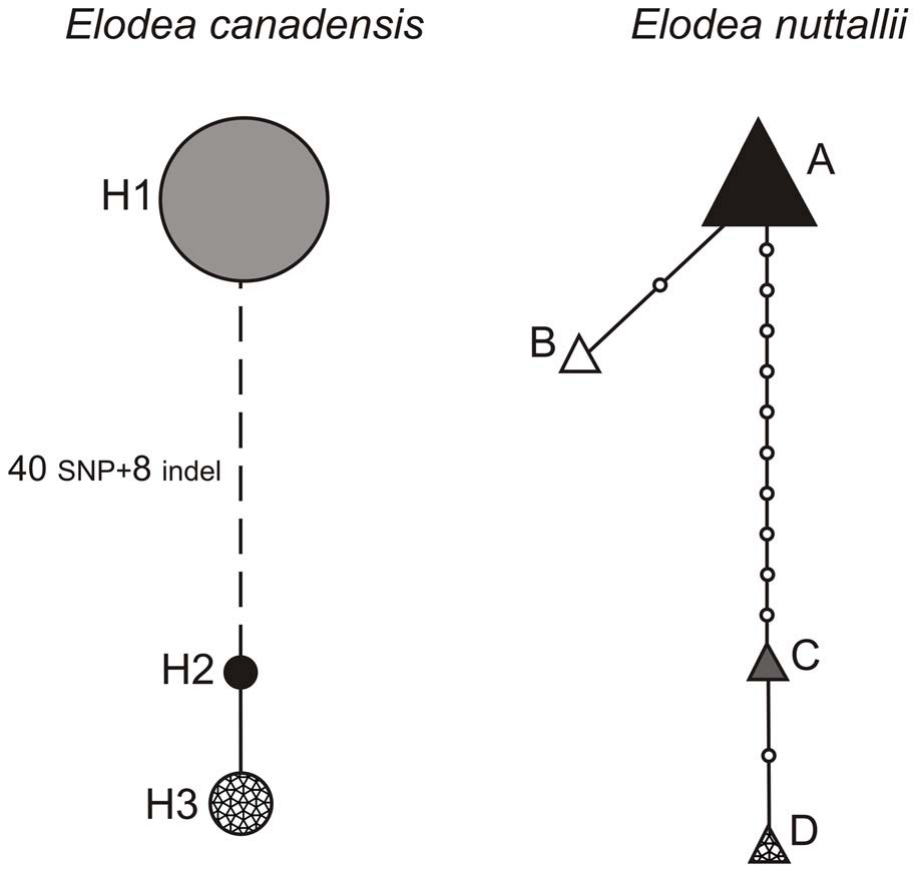
Haplotypes found in 33 *E. canadensis* and 25 *E. nuttallii* populations analyzed, based on mutation differences within 4072 bp of plastid sequence. The sizes of the circles and triangles are proportional to the number of populations included in each haplotype (H1, 29; H2, 1; H3, 1; A, 22; B 1; C, 1; D, 1). Small open circles on connecting spans indicate minimum numbers of individual mutations. Branch in dashed line represent the mutation differences between *E. canadensis* haplotypes H1 and H2, which could not be connected within the limits of parsimony (95%). These haplotypes are connected manually with observed mutations (numbers along the branch in dashed line).

N, number of samples. See Figures 1 and 2 for locations, and Figure 3 for sequence differences between haplotypes.

* USA unless otherwise specified.

1 The Finnish Museum of Natural History.

2 Oregon State University Herbarium.

3 New York Botanical Garden Herbarium.

### Sequencing and primer development

The treatment of fresh *E. canadensis* plant material and the cpDNA extraction were performed as in Huotari and Korpelainen [58], using the extraction method based on a differential chloroplast lysis using a non-ionic detergent, Triton X-100 [59]. DNA extractions were digested with restriction enzymes *Hind*III and *Eco*RI, and the resulting fragments were separated on a 1% agarose gel to determine the quality of plastid DNA. In order to further eliminate the contamination by nuclear DNA, the cpDNA extractions were treated with ATP-dependent DNase (Plasmid-Safe, Epicentre Biotechnologies), which selectively hydrolyzes linear double-stranded DNA. In order to further enrich the portion of pure cpDNA in our samples, a PCR reaction using the GenomePlex whole genome amplification kit (Sigma-Aldrich, St. Louis, MO, USA) was performed. Samples were then sequenced with 454 FLX pyrosequencer (Roche Applied Science). The sequencing run generated 98,326 raw reads of which 78,765 reads consisting of 697,024 bp were aligned into 911 contigs. These contigs reached on average 16-fold coverage over the IR regions and 4.8-fold coverage over the SSC and LSC regions. In order to find cp-related contigs, a database search was performed using the BLAST algorithm at the National Center for Biotechnology Information (NCBI). The contigs were also aligned with the reference cp genome sequence of introduced *E. canadensis* plant material (*Ec*17 in Table 1 and Figure 1) (GenBank accession number JQ310743) sequenced earlier [58]. The software Tablet [60] was used to check for the quality of contigs and to assure the order of contigs in relation to each other. Altogether we obtained 75 contigs related to the cp genome of *E. canadensis*. The contigs consisted of 3318 reads (3.4% of total reads) and were on average 1510 bp long. The contigs had a collective length of 112,193 bp, when only one copy of IRs was included; leaving 18,158 bp (13.9%) missing from the total genome. Several of the remaining gaps were too large to be closed via PCR and, therefore, all further comparative analyses of plastid sequences between the native and introduced populations of *E. canadensis* are based on this 112,193 bp-sequence of good quality (GenBank accession number for alignment will be provided).

In order to detect variable sites, the plastid sequences of *E. canadensis* collected from the native and introduced range were compared using Gap4 [61] and manually aligned using BioEdit [62] (Alignment S1). The total number and distribution of all SNPs, indels and inversions were recorded. In addition, indels consisting of mono- or dinucleotide SSRs were surveyed. We designed specific primers for the flanking sequences outside the most variable regions using Primer3 (http://frodo.wi.mit.edu/primer3/). The goal was to choose both coding and non-coding regions informative in species identification and valuable in phylogeographic analyses. Altogether, 29 primer pairs were developed containing 78 SNPs, one inversion and 24 indels, of which 15 were mono- or dinucleotide SSRs (see the total list of primer sequences and polymorphisms in Table 2). All these primers were located within single-copy regions (LSC and SSC) of the cp genome. The average coverage of single-copy regions in the pyrosequencing was 4.8. In our analyses, we only accepted polymorphic sites with 4-fold coverage or more, verified using Tablet [60], as true differences between the two plastid sequences. Additionally, variable sites with a lower coverage verified by direct sequencing with primers developed were taken into account.

**Table 2:** Characteristics, primer sequences and level of polymorphisms in 29 cpDNA marker regions developed for *Elodea canadensis*.

				Polymorphisms *E. can* FIN vs. *E. can* USA/*E. can* FIN vs. *E. nutt*/*E. can* USA vs. *E. nutt*				
Locus	Length (bp)	T_a_ (°C)	Primer sequences (5′–3′)	SNP	SSR	Indel	Variable genomic area	GenBank accession numbers
cp1125A*	395	56	F: ACATCAGATCGACGCTTTGT	–/3/3	2/2/−	−/1/1	IGS (*rps18* −*rpl20*), *rpl20*	KC812628,
			R: CTCGTGAGAATGAACTCTCCT					KC812643,
								KC812652,
								KC812673
cp1345A*	243	54	F: CCTCATCCTCAAATCAGTCC	1/1/−	1/1/1	−/1/1	IGS (*atpF* − *atpH*)	−†
			R: ACTAAGTTTGGCTTTCATGG					
cp1345B*	439	57	F: GGCCTACTTCTACACCCGATA	6/5/7	−/−/−	2/2/1	IGS (*atpI* –*rps2*), *rps2*	KC812630,
			R: CAAATTCTGACCCCGATCTT					KC812669,
								KC812704
cp1345D*	469	57	F: CGACTTTGGGCTATGGTTAGC	5/4/4	−/−/−	−/−/−	*rpoC2*	KC812640,
			R: ATCATTTTGAATCCGTTGGA					KC812647,
								KC812689
cp1345F*	393	59	F: GGCTCTCCAATTGATTGACC	5/3/8	−/−/−	−/−/ −	*rpoC2*	KC812644,
			R: AGATGCCGGATACCTCACAC					KC812668,
								KC812718
cp1689A*	598	58	F: CAAGTGCAACCAACCTCAAA	3/7/8	2/2/1	−/−/−	IGS (*atpB* − *rbcL*)	KC812648,
			R: CATAAGTCCCTCCCTACAACTCA					KC812651,
								KC812663,
								KC812709
cp1872A*	573	60	F: GGCATGAGTGAAGGAACTCG	6/12/13	1/−/1	−/1/1	IGS (*ycf4* −*cemA*)	KC812655,
			R: CCGGTGCCCAGAACAATTA					KC812664,
								KC812665
cp2381A*	400	57	F: GGTGCTTCCATGAACTGAGA	11/42/43	−/−/–	−/−/–	IGS (*ycf1* – *ndhF*), *ndhF*	KC812632,
			R: TGGTCATATAATCGGGGCTA					KC812639,
								KC812676,
								KC812690,
								KC812691
cp2493A*	562	56	F: GTCCGCTTTGCTTTATTCAT	4/4/7	−/−/–	−/−/–	*ndhD*	KC812656,
			R: ATAACTAACGCGGGACTCAA					KC812658,
								KC812672,
								KC812694,
								KC812698,
								KC812715
**Subtotal**	**4072**			**41/81/93**	**6/5/3**	**2/5/4**		
cp58A^1^	578	57	F: CTCCTCATACGGCTCAAGAA	2/8/6	−/−/–	−/1/1	intron *rps16*	KC812636,
			R: ATTCAACAAAGCAAGGGTCA					KC812659,
								KC812686
cp58B	400	58	F: CAAAATGGCAGCAACATACC	1/5/5	−/−/–	1/−/1	intron *rps16*	KC812629,
			R: GCGACTTGAAGGACATCACC					KC812684,
								KC812705
cp795A	396	56	F: TCTCGTGATTTGTATCCAAGG	−/1/1	1/1/–	−/−/–	intron *trnG*-*UCC*	KC812635,
			R: AGCTTTTCGTATTCGCTTTCT					KC812674,
								KC812716
cp796A	370	58	F: CACAATCCACCGCCTTAAT	3/8/9	−/−/–	2/2/–	IGS (*trnH*-*GUG* – *psbA*), *psbA*	KC812677,
			R: GCATGAACGTAATGCTCACA					KC812708,
								KC812711
cp797A	598	52	F: AGATTGTGACCTGGATTAAA	3/7/8	1/−/1	−/−/–	*rps3*, IGS (*rps3* – *rpl22*), *rpl22*	KC812654,
			R: CAAAGCCCGAAGAGTAATTG					KC812671,
								KC812693
cp828A	668	56	F: TAGGCCATACCCATTTCTTC	2/5/5	1/1/–	−/−/–	intron and exon *ycf3*	KC812650,
			R: AAAAGCGTTGAGGACAAAGA					KC812706,
								KC812717
cp884A	580	54	F: AGACCTAACACGATTCCAAA	3/4/5	−/−/–	1/−/1	IGS (*psbE* – *petL*), *petL*, IGS	KC812680,
			R: CTCATCACCAGTTACACAATGAA				(*petL* – *petG*)	KC812682,
								KC812688
cp1110A	431	58	F: AAGCAGAAACATAGATGCACTCC	4/11/11	−/−/–	1/1/1	IGS (*ndhC* - *trnV*-*UAC*)	KC812679,
			R: GCATGATGAAATGGAACGAA					KC812700,
								KC812710
cp1110B	557	56	F: CGAATCCATGGAGTAAGACA	−/1/1	1/−/1	1/1/–	*trnV-UAC*, IGS (*trnV*-*UAC* –	KC812660,
			R: AGCAGAACAATCACAAGAGC				*trnM*-*CAU*), *trnM-CAU*, IGS	KC812699,
							(*trnM*-*CAU* – *atpE*)	KC812703
cp1345C	495	57	F: ATGACCAAAATGGACTCCTG	2/7/5	−/−/−	−/1/1	*rps2*, IGS (*rps2* – *rpoC2*),	KC812645,
			R: AGTACACCGCTCAAAGCAAC				*rpoC2*	KC812661,
								KC812712
cp1708A	440	56	F: AATAGCCAACTGGATCGAA	1/1/2	1/1/−	−/−/-	IGS (*rpoB* - *trnC*-*GCA*)	KC812687,
			R: TTACACGGATACGAGTCAGG					KC812707,
								KC812714
cp1708C^2^	373	56	F: TGGATTGGTCGAAATTGATA	1/4/5	−/−/−	1/1/−	IGS (*petN* - *psbM*)	KC812634,
			R: CCGAGTCTTAATGAAATGGAA					KC812649,
								KC812678
cp1708E	466	56	F: TGCTATTCTTTTACGCCACA	1/2/2	1/1/1	−/−/−	IGS (*trnT*-*GGU* – *psbD*)	KC812642,
			R: AACGGGTTTCGAAGATACAA					KC812685,
								KC812713
cp1708F	385	56	F: GGCTCTCCAATTGATTGACC	¼/3	−/−/−	−/−/−	IGS (*rps14* - *psaB*), *psaB*	KC812646,
			R: AGATGCCGGATACCTCACAC					KC812667,
								KC812692
cp1872C	298	59	F: CTTCGCATCCGTTATTTTGG	1/3/2	1/1/-	−/1/1	*petA*, IGS (*petA* – *psbJ*)	KC812633,
			R: TTTGCCTCCCATACCCATTA					KC812666,
								KC812695
cp1970A	699	57	F: CAGTCGCACTTTGGTTAGGT	1/1/2	1/−/1	−/−/−	*petD*, IGS (*petD* - *rpoA*), *rpoA*	KC812657,
			R: ATGGACAAATGGGAGTTTCA					KC812696,
								KC812697
cp2320A	299	57	F: GCCGACTTGATATTGGCATT	1/1/2	1/−/1	−/−/−	intron and exon (*petB*)	KC812638,
			R: TGTTGACATGAGGAGGAACA					KC812653,
								KC812701
cp2328A	570	57	F: AATCCTCGTGTCACCAGTTC	4/10/10	−/−/−	−/−/−	IGS (*trnF*-*GAA* – *ndhJ*)	KC812641,
			R: TTTCTCCTCCGTTCTAGCTG					KC812670,
								KC812702
cp2367A	600	50	F: TCCTCTCGAACCATACTAA	1/23/23	−/1/1	−/−/−	*rps11*, IGS (*rps11* – *rpl36*)	KC812631,
			R: GGAAGCACTAATGTAAGTCA					KC812675,
								KC812681
cp2381B	499	56	F: GGACCCATAAAGAATGTATGC	4/4/5	−/−/−	−/−/−	*ndhF*	KC812637,
			R: CGACGGATATTTCCATGTTC					KC812662,
								KC812683
**Total**	**11,464**			**77/191/205**	**15/11/9**	**9/13/10**		

IGS, Intergenic spacer.

* Nine loci used in phylogeographic analysis.

† Sequences too short for GenBank, more information from the authors (tea.huotari@helsinki.fi).

1 Additional inversions: 1/–/1.

2 Additional inversions: −/1/1.

Sequence differences are indicated between the two *E. canadensis* plastid sequences FIN and USA and between both *E. canadensis* plastid sequences and *E. nuttallii* plastid sequence (*En*13, Table 1).

### Molecular methods

For the phylogeographic analyses, genomic DNA was extracted from dried leaf tissue of 70 *E. canadensis* samples collected from 16 native and 17 introduced populations, and 25 *E. nuttallii* samples collected from 22 native and 3 introduced populations. The DNA was extracted using a commercial kit (E.Z.N.A. Plant DNA Mini Kit, Omega Bio-Tek) following the manufacturer′s protocol. All these samples were analyzed using nine, potentially highly variable markers comprising 4072 bp (see Table 2). Additionally, we used all the designed 29 primer pairs comprising 11,464 bp (Table 2) to compare the level of intraspecific variation between the two *E. canadensis* plastid sequences (*Ec*14, hereafter USA and *Ec*17, hereafter FIN in Table 1) to that of interspecific variation between *E. canadensis* and *E. nuttallii* (*En*1, Table 1) plastid sequences. All PCR amplifications were performed in a total volume of 20 µl containing about 5 ng genomic DNA, 13 µl ddH_2_O, 1× reaction buffer including 1.5 mM MgCl_2_, 1 mM dNTP mix, 0.6 µl Dynazyme II DNA Polymerase (Finnzymes) (2 U/µl) and 5 pmols of both primers. The thermal cycler was programmed for 5 min denaturation at 94°C, followed by 35 cycles of denaturation at 94°C for 30 s, annealing at 50–60°C for 30 s, and elongation at 72°C for 1 min, and a final 10 min extension at 72°C. The amplification products were separated on 1.2% agarose gel, and clear bands were excised and purified with a commercial kit (E.Z.N.A Gel Extraction Kit, Omega Bio-Tek). Purified products were sent for DNA sequencing (Macrogen Inc., South Korea). Alignments of sequences were adjusted manually using BioEdit [62] and trimmed to the same length.

### Data analysis

The sequences obtained using 29 cpDNA markers comprising 11,464 bp (Table 2) were compared using BioEdit [62] to detect the level of variation between native and introduced individuals of *E. canadensis*, and between *E. canadensis* and *E. nuttallii*. In addition, all collected *E. canadensis* and *E. nuttallii* specimens were characterized for cpDNA haplotypes based on the nine selected cpDNA regions comprising 4072 bp (Table 2). These regions were chosen to represent both coding and non-coding cp genome regions, and to contain SNP-, SSR- and indel-variation based on the initial comparison between the two *E. canadensis* plastid sequences USA and FIN. The geographical distributions of the cpDNA haplotypes were analyzed in native and introduced ranges of *E. canadensis* and *E.nuttallii*. Haplotype networks from the sequence data were constructed using the network building software TCS 1.2.1 [63], which uses statistical parsimony and the genealogical reconstruction algorithms of Templeton et al. [64]. We treated indels as a fifth state [65], [66], and coded each indel as a single mutational event [67]. One *E. canadensis* haplotype (H1) could not be connected by TCS and is linked to other haplotypes with a dashed line in Figure 3. This haplotype was connected manually with the observed mutational differences (this is not intended to connect the network but used to demonstrate minimum distances between unconnected haplotypes).

## Results

**Figure 4:**
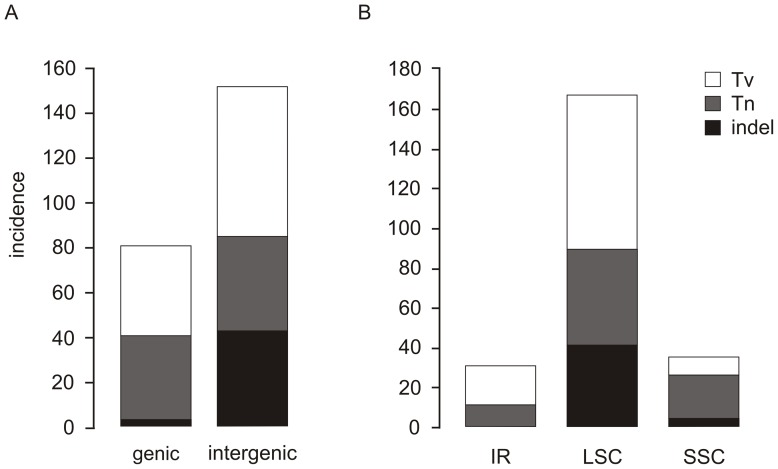
Classification of mutational differences between the two *Elodea canadensis* chloroplast genomes. Classifications of single nucleotide polymorphisms (SNPs) and insertion/deletion (indel) differences between the two *Elodea canadensis* plastid sequences (FIN and USA), based on 112,193 bp. A: genic and intergenic regions, B: inverted repeat (IR), large single-copy (LSC) and small single-copy (SSC) regions. Tv, transversion; Tn, transition; indel, insertion/deletion.

The 112,193 bp of cpDNA sequence compared between native (USA) and introduced (FIN) *E. canadensis* plastid sequences contained altogether 235 variable sites (186 SNPs, 47 indels and two inversions) (Table 3). Most variation occurred within the non-coding regions of LSC (Figure 4). The substitution rate within the single-copy regions was 0.00181 per nucleotide, while the rate in the inverted repeat region was 0.00119. The observed ratio (R) of transitions (Tn) to transversions (Tv) was 0.74. Of the 186 SNPs detected, 79 were Tn and 107 were Tv (Table 3). In all, 109 SNPs were located in non-coding regions while 77 SNPs were found within coding regions. Of these, 42 SNPs were nonsynonymous and 35 were synonymous substitutions (Table 3, Figure 4).

**Table 3:** Summary of differences detected between the two plastid sequences of *Elodea canadensis* collected from the native and introduced range, based on 112,193 bp.

Gene	In/Del	Inversion	1Tn	2Tv	Nonsyn	Total
*accD*			1			1
*atpE*				1		1
*atpI*			1			1
*cemA*				1		1
*matK*				1		1
*ndhA*			2		1	2
*ndhD*			3	1	2	4
*ndhF*			5	1	4	6
*petA*				2		2
*petN*				1		1
*psbB*			1		1	1
*psbC*				2		2
*psbH*			1			1
*psbJ*			1			1
*rpl22*				1	1	1
*rpoA*			1	1	1	2
*rpoB*			1			1
*rpoC1*	1		1	1	1	2
*rpoC2*			7	11	12	18
*rps2*			1		1	1
*rps3*			1	1		2
*trnG-GCC*			1			1
*rpl23*	1					1
*rrn23*			2	1		3
*ycf1*	1	1	2	8	9	12
*ycf2*	1		5	6	9	12
Subtotal coding	4	1	37	40	42	82
Subtotal noncoding	43	1	42	67	-	153
**Total**	**47**	**2**	**79**	**107**	**42**	**235**

1 Tn, Transition.

2 Tv, Transversion.

A total of 47 indels were identified between the USA and FIN plastid sequences, of which four were located within coding regions (*rpoC1*, *rpl23*, *ycf1* and *ycf2*) (Table 3) and four in introns (*trnG-GCC*, *ycf3*, *petB* and *rpl16*). The three longest indels were 141 bp (between *ycf2* and *trnL-CAA*), 38 bp (between *ndhC* and *trnV-UAC*) and 23 bp (between *petN* and *psbM*) in length, while the rest of the indels were 10 bp or less in length. Altogether, 28 indels located in LSC were mono- or dinucleotide repeat regions (SSRs) showing length polymorphism. Of them, 25 SSRs were mononucleotide repeats (4×A, 19×T, 1×C, 1×G) with an average length of 8.3 bp, while three were dinucleotide repeats (TA/AT) with an average length of 13 bp (Table 4). Two structures were interpreted as minute inversions, typical for non-coding regions of cpDNA [68], [69]. The flanking sequence outside the inverted regions was explored, and a hairpin structure was found from the inversion located in *rps16* intron, consisting of a stem of eight bp and a loop of three inverted bp (GTA/TAC). Another potential inversion (CG/GC) was found in *ycf1* gene, but it did not form a hairpin.

**Table 4:** Chloroplast microsatellites (SSRs) showing length polymorphism between the two plastid sequences of *Elodea canadensis* collected from native (USA) and introduced (FIN) range, based on 112,193 bp.

Location	USA	FIN	SSR start^1^	SSR end
intron (*trnG-GCC*)	(A)_9_	(A)_10_	9629	9638
IGS	(T)_9_	(T)_7_	13777	13785
IGS	(T)_6_	(T)_7_	14114	14120
*rpoC1*	(T)_4_	(T)_7_	23797	23803
IGS	(A)_10_	(A)_9_	27738	27747
IGS	(T)_12_	(T)_11_	28231	28242
IGS	(A)_6_	(A)_7_	32825	32831
IGS	(T)_8_	(T)_9_	33664	33672
IGS	(C)_4_	(C)_5_	38106	38110
intron (*ycf3*)	(T)_9_	(T)_8_	44532	44540
IGS	(AT)_7_	(AT)_6_	47164	47177
IGS	(T)_13_	(T)_9_	47854	47866
IGS	(T)_10_	(T)_9_	49242	49251
IGS	(T)_8_	(T)_10_	53899	53903
IGS	(T)_9_	(T)_16_	56245	56260
IGS	(T)_8_	(T)_6_	56368	56375
IGS	(T)_7_	(T)_6_	62082	62088
IGS	(T)_7_	(T)_4_	64758	64764
IGS	(T)_11_	(T)_12_	65835	65846
IGS	(T)_6_	(T)_7_	67023	67029
IGS	(T)_10_	(T)_8_	70048	70057
IGS	(TA/AT)_5_	(TA/AT)_6_	70071	70082
IGS	(T)_8_	(T)_7_	75626	75633
intron (*petB*)	(G)_7_	(G)_8_	77248	77255
IGS	(T)_8_	(T)_11_	79490	79500
IGS	(A)_7_	(A)_8_	82761	82768
intron (*rpl16*)	(TA)_9_	(TA)_6_	83499	83516
IGS	(T)_9_	(T)_10_	86262	86271

1 Numbering according to cp genome of *E. canadensis* collected from introduced range (GenBank accession number JQ310743).

29 cpDNA markers comprising 11,464 bp (Table 2) revealed 102 variable sites (77 SNPs, 24 indels and one inversion) between the two *E. canadensis* plastid sequences (USA and FIN), 216 variable sites (191 SNPs, 24 indels and one inversion) between *E. canadensis* (FIN) and *E. nuttallii* (*En*1) plastid sequences and 226 variable sites (205 SNPs, 18 indels and two inversions) between *E. canadensis* (USA) and *E. nuttallii* (*En*1) plastid sequences (Table 2). We discovered on average 61% more SNPs between the two *Elodea* species than between the two *E. canadensis* plastid sequences, while the number of indels was 10% larger within *E. canadensis* comparison. Both inversions found between *E. canadensis* and *E. nuttallii* formed a hairpin structure similar to the one found between the two *E. canadensis* cp genome sequences. One inversion was the same as between *E. canadensis* cp genomes (GTA/TAC) located in *rps16* intron, and the other inversion was located in non-coding region between *petN* and *psbM* genes, consisting of a stem of 12 bp and a loop of three inverted bp (TAT/ATA). On the whole, the level of variation between the two *Elodea* species was 53% higher than that within *E. canadensis* (Table 2).

In the phylogeographic analysis using nine cpDNA markers comprising 4072 bp (Table 2) we found three *E. canadensis* and four *E. nuttallii* haplotypes (Figures 1 and 3) (Alignment S2). The haplotype network constructed indicates that *E. canadensis* haplotype H1 is very divergent from haplotypes H2 and H3, as it could not be connected within the limits of parsimony (95%) (Figure 3). Haplotypes H1 and H2 were separated by 48 mutations including eight indels, while haplotypes H2 and H3 were separated by just one mutation (Figure 3). In *E. nuttallii*, haplotypes A and B, separated by two mutations, were divergent from haplotypes C and D, which differ from each other by two mutations (Figure 3). Only a single haplotype was found in the introduced range in each of the two species. These haplotypes H1 (*E. canadensis*) and A (*E. nuttallii*) were also widespread in the native range, covering the majority of native populations analyzed (H1 88% and A 86%) (Figure 1). The largest divergence between *E. canadensis* haplotypes was about four times larger than the largest divergence between the *E. nuttallii* haplotypes.

## Discussion

### Plastid sequence polymorphisms

Most intraspecific studies of cpDNA variation have utilized a limited number of markers from a few selected gene regions. Only few studies have examined intraspecific variation in the whole cp genome sequence [70]–[72]. We were now able to investigate variation in an 112,193 bp (86.1%, only one IR included) region of two *E. canadensis* plastid sequences and found a total of 235 variable sites. In general, the mutation rate of SNPs (often about 10^−8^–10^−9^) is low compared to SSRs (about 10^−4^). On average, one SNP can be expected every 500-1000 bp in coding regions and every 200-500 bp in non-coding regions [73]. The evolutionary rate of cpDNA is only half of that in nuclear DNA in plants [26] and, therefore, also the incidence of SNPs in cp genome is expected to be lower. We found on average one SNP every 863 bp in coding and every 420 bp in non-coding regions. The total frequency was one SNP every 603 bp. Based on these figures, the frequency of SNPs between *E. canadensis* cp genomes is higher than expected. The SNP rate in the IR region was five times lower than that in the single-copy regions. This result is consistent with a previous report indicating that the synonymous substitution rate of IR region is roughly five times lower than that of the single copy regions [74].

In our earlier study [58] we found a total of 83 mono- and 41 dinucleotide repeats of 8 bp or greater in a survey of the whole *E. canadensis* (FIN) cp genome. In this study, we identified polymorphic SSRs between *E. canadensis* USA and FIN plastid sequences and found 25 mono- and 3 dinucleotide repeats showing length polymorphism (Table 4). All polymorphic SSRs were located in LSC, whereas in the previous survey, 77.2% of SSRs were found in LSC [58]. Nine polymorphic SSRs discovered in this survey were shorter than 8 bp in both plastid sequences, and therefore they were excluded from the previous search for longer repeat regions. We also found two inversions as a part of a hairpin loop. These kinds of small inversions flanked by inverted repeats are ubiquitous in the cp genomes of angiosperms [69], and have been found between species and genera [68], [69] as well as within species, for example in *Lolium* [71] and *Abies* [75].

### Chloroplast DNA phylogeography

The number of haplotypes in an invasive range is a function of many factors, including the number of introductions, the size of each introduction, the population structuring in the native range, and subsequent drift and selection pressures that occur after the introduction [17]. In our phylogeographic analysis, only a single haplotype was found in the introduced range in both species (Figure 2). These haplotypes H1 (*E. canadensis*) and A (*E. nuttallii*) were also widespread in the native range, covering the majority of native populations analyzed (Figure 1). Therefore, we were not able to identify either the geographic origin of the introduced populations or test the hypothesis of single versus multiple introductions. The result could indicate one introduction event, but multiple introductions of the same haplotype are just as possible. The introduced populations of *E. canadensis* have been previously studied using AFLP and SSR markers. The analysis using AFLP markers detected low levels of genetic diversity among *E. canadensis* populations and suggested one introduction event or multiple introductions of similar genotypes to New Zealand [76]. An analysis performed using SSR markers revealed a moderate level of variation in introduced populations of *E. canadensis* [77], [78]. However, the mutation rate of SSR markers is high [73], and these markers were developed in order to discover variation patterns in the introduced *E. canadensis* populations. Therefore, it is not surprising that the markers were polymorphic and revealed multiclonal populations even though only one cpDNA haplotype was found from the introduced range.

Despite the limited variation, the data presented here provides interesting information of the genetic patterns in the native and introduced ranges of *E. canadensis* and *E. nuttallii*. The cpDNA haplotype homogeneities at introduced range are most likely attributed to bottleneck followed by a single introduction event or few introductions of similar haplotypes. This pattern is further supported by the vegetative regeneration, combined to the relatively recent and fast expansions of both species in their introduced ranges. On the other hand, the divergence between *E. canadensis* haplotypes at the native range was surprisingly high, as haplotype H1 differed from haplotypes H2 and H3 by 40 SNPs and eight indels. In comparison, the largest difference between *E. nuttallii* haplotypes was 11 SNPs and one indel. The diverged haplotypes were also geographically clustered in both species, although, a more detailed sampling would be necessary to get further support for the results presented here (Figure 1). Overall, the level of variation between the *E. canadensis* plastid sequences (USA and FIN), was constantly higher than what has been discovered in other intraspecific comparisons in angiosperms [70]–[72]. On the other hand, the level of interspecific variation between *E. canadensis* and *E. nuttallii* was still 53% higher than intraspecific variation within *E. canadensis* based on 11,464 bp of cpDNA. The large divergence between *E. canadensis* haplotypes let us hypothesize the evolutionary event behind this phenomenon.

The divergent haplotypes could be explained by chloroplast capture, where the cytoplasm of one species is replaced by that of another species through hybridization or introgression, as reported to occur in natural ecosystems [79], [80]. Chloroplast capture can occur in species with sympatric distribution and reproductive compatibility, and the process is facilitated by weak reproductive barriers between species [81]. There is some support for naturally occurring hybrids between *E. canadensis* and *E. nuttallii* [42]. However, most of the variable sites found between *E. canadensis* plastid sequences were not detected in the *E. nuttallii* plastid sequence, indicating that hybridization between these species could not explain the large divergence between *E. canadensis* haplotypes. Interestingly, the distributions of *E. canadensis* and *E. bifoliata* would enable a hybrid zone allowing the chloroplast capture event involving the largely parapatric *E. bifoliata*. However, an extensive survey of genetic characteristics of *E. bifoliata* would be needed to firmly retrace the possibility of chloroplast capture.

Another reason for this high haplotype divergence in E. canadensis might be species mis-identification, but this is unlikely. We used the ITS region for species identification in all specimens and did not find any contradictory results. This region differs between *E. canadensis* and *E. nuttallii* by nine indels and 27 point mutations [56]. Therefore, it may be expected to differ also between *E. canadensis* and *E. bifoliata*, which are morphologically more differentiated. The ITS regions of morphologically similar *Hydrilla verticillata* (GenBank accession number AY870353.1) and *Egeria densa* (GenBank accession number AY870360.1) are clearly different from those of *E. canadensis* and *E. nuttallii* leaving no risk of confusion. In addition, neither *E. canadensis* nor *E. nuttallii* are usually confounded with other *Elodea* species based on morphology. On the other hand, we found several variable sites between *E. nuttalli* haplotypes as well, and the level of variation between *E. canadensis* and *E. nuttallii* plastid sequences was still 53% higher than that between the two *E. canadensis* plastid sequences. In addition, while ITS sequences of *E. nuttallii* have been found to be highly homologous, those of *E. canadensis* include several polymorphic sites [56]. Therefore, the possibility of a higher level of naturally occurring variation in both nuclear and cp genomes of *E. canadensis* cannot be ruled out based on our results.

### Chloroplast genome as a source of genetic markers

*Elodea canadensis* and *E. nuttallii* are morphologically very variable and, therefore, difficult to discriminate [33], [47], [55]. Also in this study, 38% of all sampled populations, were misidentified when using morphological characteristics. Even though the nuclear ITS region has been reported as a reliable identification tool to discriminate *E. canadensis* and *E. nuttallii* [56], additional molecular markers for species identification are useful especially for monitoring purposes and for early detection of new invasions. The cpDNA markers developed in this study are valuable tools for species identification between *E. canadensis* and *E. nuttallii*. Of these markers, loci cp2367A (*rps11*, IGS (*rps11* – *rpl36*) differed by at least 20 point mutations and 2381A (IGS (*ycf1* – *ndhF*), *ndhF*) by at least 40 point mutations between the two *Elodea* species (Table 2). In addition, loci cp1110A (IGS (*ndhC* – *trnV*-*UAC*), cp1872A (IGS (*ycf4* – *cemA*) and cp2328A (IGS (*trnF*-*GAA* – *ndhJ*) differed by at least ten point mutations (Table 2). Most variable loci between the two *E. canadensis* cpDNA sequences were cp1345B (IGS (*atpI* – *rps2*), *rps2*), cp1872A and cp2381A (Table 2). All the cpDNA markers developed in this study are potentially variable novel markers applicable to population level, phylogenetic and phylogeographic research in *Elodea* species and most likely among other angiosperms as well.

## Supporting Information

Alignment S1 **Alignment of the two** ***Elodea canadensis*** **chloroplast genomes (USA and FIN) in nexus format.** GenBank accession number for FIN sequence is JQ310743.
(NEX)

Alignment S2 **Chloroplast DNA sequence polymorphisms of three** ***Elodea canadensis*** **haplotypes (H1–H3) and four** ***E. nuttallii*** **haplotypes (A–D) revealed using nine cpDNA loci.** For primer information and genomic regions amplified, see Table 2.
(TXT)
